# Obituary

**Published:** 1868-05

**Authors:** 


Obituary.—Died in Dunkirk, N. Y., May 8th, John C. Matteson, M. D., aged 38 years.

At a meeting of the Physicians of Dunkirk, appropriate resolutions were passed expressing their sense of loss and their sympathy for the family of the deceased.

